# Shared larval rearing environment, sex, female size and genetic diversity shape *Ae*. *albopictus* bacterial microbiota

**DOI:** 10.1371/journal.pone.0194521

**Published:** 2018-04-11

**Authors:** Guillaume Minard, Florence-Hélène Tran, Van Tran Van, Corentin Fournier, Patrick Potier, David Roiz, Patrick Mavingui, Claire Valiente Moro

**Affiliations:** 1 Université de Lyon, Lyon, France, Université Lyon 1, Villeurbanne, France; CNRS, UMR 5557, Ecologie Microbienne, Villeurbanne, France, INRA, UMR1418, Villeurbanne, France; 2 Metapopulation Research Center, Department of Biosciences, University of Helsinki, Helsinki, Finland; 3 Infectious Diseases and Vectors: Ecology, Genetics, Evolution and Control, IRD (Institut de Recherche pour le Développement), Montpellier, France; 4 Université de La Réunion, CNRS 9192, INSERM U1187, IRD 249, Unité Mixte Processus Infectieux en Milieu Insulaire Tropical (PIMIT), Plateforme Technologique CYROI, Sainte-Clotilde, La Réunion, France; Universidade Federal do Rio de Janeiro, BRAZIL

## Abstract

The Asian tiger mosquito *Aedes albopictus* became of public health concern as it can replicate and transmit viral and filarial pathogens with a strong invasive success over the world. Various strategies have been proposed to reduce mosquito population's vectorial capacity. Among them, symbiotic control of mosquito borne disease offers promising perspectives. Such method is likely to be affected by the dynamics of mosquito-associated symbiotic communities, which might in turn be affected by host genotype and environment. Our previous study suggested a correlation between mosquitoes’ origin, genetic diversity and midgut bacterial diversity. To distinguish the impact of those factors, we have been studying the midgut bacterial microbiota of two *Ae*. *albopictus* populations from tropical (La Réunion) and temperate (Montpellier) origins under controlled laboratory conditions. the two populations experienced random mating or genetic bottleneck. Microbiota composition did not highlight any variation of the α and β-diversities in bacterial communities related to host’s populations. However, sizes of the mosquitoes were negatively correlated with the bacterial α-diversity of females. Variations in mosquito sex were associated with a shift in the composition of bacterial microbiota. The females’ mosquitoes also exhibited changes in the microbiota composition according to their size and after experiencing a reduction of their genetic diversity. These results provide a framework to investigate the impact of population dynamics on the symbiotic communities associated with the tiger mosquito.

## Introduction

The Asian tiger mosquito *Aedes albopictus* has been recently considered as one of the “100 World’s worst invasive alien species” (Global Invasive Species Database) [1]. Originating from Asia, *Ae*. *albopictus* has spread over 5 continents during the last decades [2]. Though the mosquito shows a poor active dispersal ability by flight (less than 300 m.), passive dispersal in goods (tyres or Lucky Bamboo) due to increasing global trade has been largely involved in its spread [3]. *Ae*. *albopictus* has also been considered as one of the most important disease vectors and has already been identified as a potentially competent vector for more than 22 viruses in the laboratory and being responsible for the epidemic transmission of chikungunya, dengue and zika [4].

Viruses acquired by the mosquito through blood meal need to accomplish replication cycles inside the insect before being transmitted to a vertebrate host. During this extrinsic incubation period, ingested virus particles first reach the insect midgut and then cross the epithelial barrier to finally reach the salivary gland through the hemolymph [5]. At the first step of replication, the viruses need to reach the apical pole of the midgut epithelial cells to replicate. This step has been demonstrated to be costly for the viral population and consequently represents a strong bottleneck [6,7]. Several factors have been suggested to affect the viral population within the gut such as (i) unfavorable conditions (epithelial cell receptivity, peritrophic matrix, lytic enzymes), (ii) mosquito’s immunity and (iii) mosquito’s microbiota [5,8–10]. The latter one could directly impact viral replication by the production of antiviral factors or barrier effect, but could also induce several indirect effects such as immune priming [11].

Because of those properties several bacteria colonizing midguts or other tissues have been suggested as potential tools to control vector capacity of the mosquitoes [12,13]. As an example, either *Chromobacterium* Csp_P or *Wolbachia w*MelPop-CLA has shown a significant ability to interfere with dengue virus [14,15]. Other applications called paratransgenesis rely on the colonization of mosquito populations by genetically engineered bacteria [16,17]. The symbiotic bacteria from the genus *Asaia* sp. and *Pantoea* sp. have been largely proposed for such applications due to their ability to colonize stably a wide range of mosquitoes [18–20]. However, recent advances showed that ecological interactions between symbionts could also shape the microbial communities of mosquitoes [21,22]. Indeed, the bacterium *Asaia* which is stably associated with *Anopheles* sp. impedes the colonization of this mosquito by the endosymbiotic bacteria *Wolbachia* [22]. Therefore, understanding the factors shaping the midgut microbiota dynamics should be one of the first steps to disentangle their use in symbiotically-modified mosquitoes.

Several descriptive studies have already provided scarce but useful information about the main factors driving the mosquito midgut intestinal communities’ composition. Among those, nutrition, development and sex might have a strong influence [12]. Studies based on different *Ae*. *albopictus* populations highlighted strong dominance and prevalence of the endosymbiotic bacteria *Wolbachia*, being doubly-infected with strains *w*AlbA and *w*AlbB [23,24]. However, these symbionts are mainly located in reproductive organs and poorly infect epithelial cells of mosquito midguts [24–26]. Whole microbiota composition of *Ae*. *albopictus* was also shown to be affected by the nutritional behavior of the mosquito. Indeed blood and sugar fed females harbor distinct bacterial communities [27]. Nutritional behavior might also be responsible for microbiota differences between males and females as only the latter sex needs blood in order to accomplish its gonotrophic cycle [28]. On top of those factors involved in symbiont-hosts associations, several studies reported a shift in the microbiota composition of different mosquito populations [24,28]. However, those field-based studies were mainly correlative and were not designed to disentangle the impact of habitat quality or mosquito genetic background. During our recent field study, we observed a significant correlation between mosquitoes’ genetic diversity and midgut microbiota diversity [24]. Genetic diversity reductions have been consistently observed in invasive populations. The factors responsible for the reduction of genetic diversity in *Ae*. *albopictus* have never been deeply investigated, several hypotheses have been proposed such as a founder effect, a genetic drift associated with the isolation of the new local population or a Wahlund effect (previously reviewed [29]). Recent studies on *Ae*. *albopictus* vector capacity have highlighted a strong genotype x environment interaction in regulating the ability of viruses to get replicated and transmitted [30]. Coordinated changes in the host genetic diversity and microbiota diversity could therefore be involved in the high competence level estimated in invasive populations of *Ae*. *albopictus* [31].

To test whether different populations collected in distant locations with various levels of genetic diversity would harbor differences in their midgut symbiotic communities, we have designed a controlled experiment excluding the impact of environmental variables. This experimental design aimed to induce a genetic bottleneck (inbred lines) in two distinct populations. Those inbred lines were compared to control lines in which no genetic bottleneck was induced. Cohort densities (number of individuals) as well as individual factors (size and sex) were also recorded.

## Material and methods

This article does not contain any studies with human participants or animals (invertebrates are excepted from legal ethical concerns) performed by any of the authors. The defibrinated rabbit blood was purchased from a slaughterhouse approved by the French ministry of agriculture (authorization number FR 42.021.002 CE).

### Mosquito rearing

**Fig 1:**
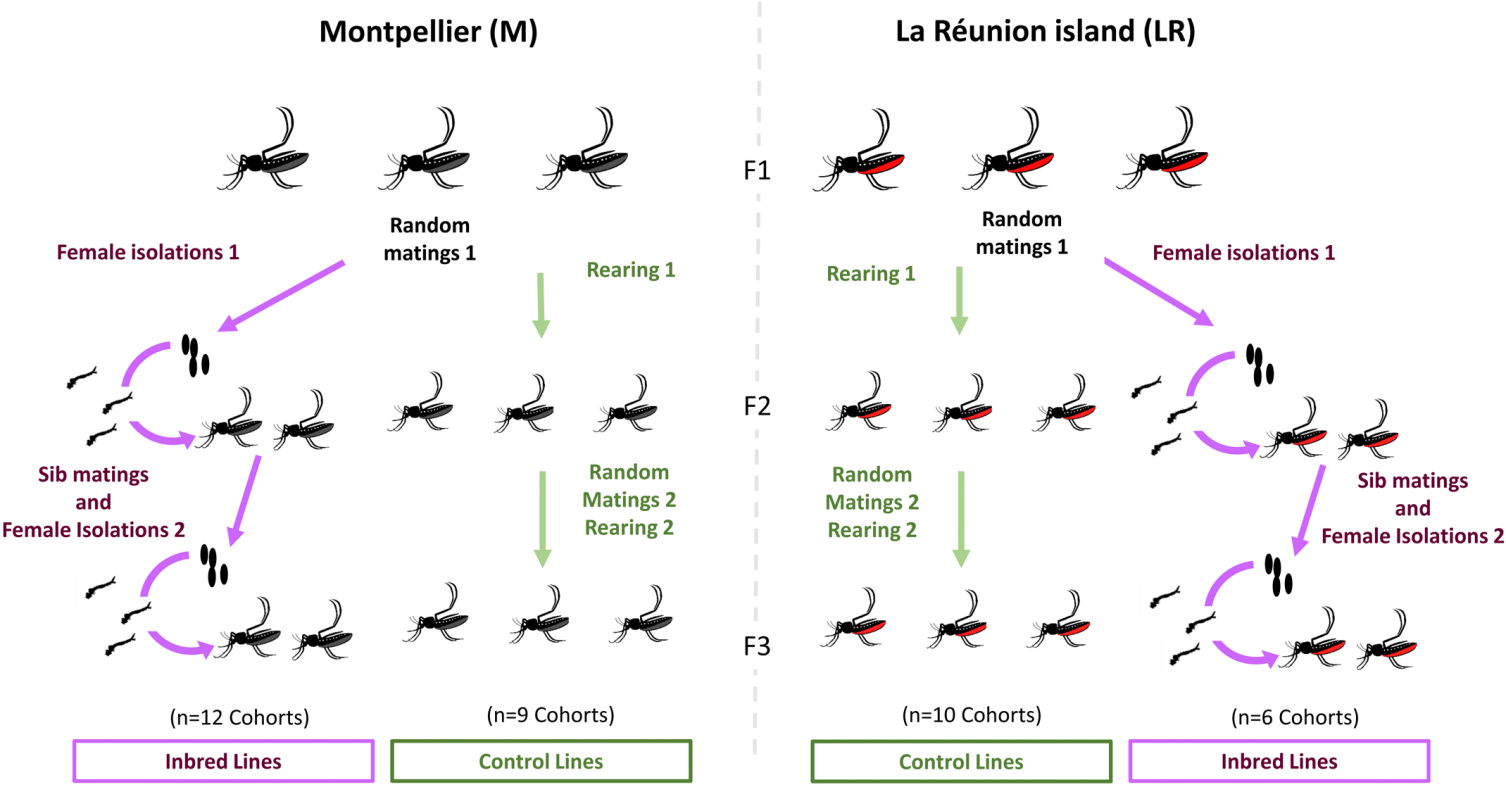
Experimental design. The F1 generation correspond to >100 individuals from non-inbred populations collected in La Réunion (LR) or collected and reared for two generations in Montpellier (M). F3 inbred lines are the progeny of sib mated F2 that have been obtained from egg clutches of an isolated female of the F1 generation. Each inbred cohort is the progeny of a single sib mated F2 female and their density varies according to the number of individuals that hatched from the same egg clutches. F3 control lines are derived from at least 3 eggs clutches merged in the same tubes and derived from the same populations after random mating of 50 individuals during two generations (F1 and F2). Control lines have been merged and reared at two larvae cohort density during the F3 generation (10 or 20 individuals).

Eggs were obtained from 3 independent ovitrap containers in Montpellier (south-east of France mainland) in October 2014 and in Saint Denis—la Réunion (French island in the south west of the Indian Ocean) in February 2015. The individuals from Montpellier were reared in the Institute for Research on the Development during two generations and allowing for random mating among >1000 individuals from the 3 containers. Eggs from F2 Montpellier and F0 La Réunion were then reared in a Bio-Safety Level 2 insectary at the University of Lyon (France) following a cycle of 18h/6h (Day/night). The Larvae were reared in dechlorinated water at 26°C and fed with a mix of 25 mg.100mg^-1^ dehydrated Yeast (Biover, Belgium) and 75 mg.100mg^-1^ dehydrated Fish food (Tetra, France). Once they pupated, they were transferred into cages until their emergence. Adult mosquitoes were reared in growth chambers (Panasonic, Japan) at 28°C and fed with a solution of 10% sucrose. The next steps of the protocol are described in the Fig 1. Adults from both populations (Montpellier, La Réunion) were first mass reared in two different cages containing more than 100 individuals and allowing for random mating. Mated females were fed 2 times per generation with defibrinated rabbit blood (Bergerie de la Combe aux loups, France) supplemented with ATP 10 mM (Life Technologies, USA) and using the Hemotek system (Hemotek medical inc., U.S.A.). Females from the cages could lay eggs on 100 ml dechlorinated water containers. The mass rearing process was repeated for two generations and the progeny was reared in cages of 50 individuals. In parallel, 10 females from the two populations (Montpellier, La Réunion) were isolated from the first generation after their first blood meal and could lay eggs in individual's water containers. Their progeny was then reared in individual water containers and then transferred in cages. A total of 20 blood-engorged females were individually isolated from each cage to a new cage. Each female was isolated with a kin male to ensure inbreeding. Eggs were collected and reared until emergence following previous conditions. The control larvae were reared in similar conditions than the inbred lines and no control was performed on mothers’ partner choices. Each inbred cohort was originating from a single sib mated F2 and eggs hatched per cohort from inbred lines was variable. To limit the differences in microbiota due to density dependent effects, two densities of larval populations were also prepared for the control lines (10 individuals and 20 individuals per cohort). From 6 to 12 cohorts of individuals from the same line and origin were reared in separated containers. Inbred lines correspond to full sib inbred larvae and control lines correspond to non-inbred larvae coming from three independent egg clutches (Fig 1, Table 1). Adults emerging from the containers were collected daily without any feeding to limit a colonization of the gut by food related transient bacteria. After sexing, the mosquito individuals were stored in a -20°C freezer until processing. A total of 313 individuals were generated and used for further experiments (see Table 1 for more details).

**Table 1:** Samples used in the study.

Population Origin	Lines categories	Cohorts	Number of F3 Larvae per cohort	Number of individuals analysed		
				Males	Females	Total
La Réunion Island (LR)	Inbred lines	LR11	3	2	1	3
		LR12	6	1	3	4
		LR13	29	4	6	10
		LR14	13	5	5	10
		LR15	28	7	6	13
		LR32	31	6	5	11
	Control lines	LRB21	10	4	4	8
		LRB22	10	3	2	5
		LRB23	10	3	4	7
		LRB24	10	5	4	9
		LRB31	20	4	4	8
		LRB32	20	6	4	10
		LRB33	20	5	2	7
		LRB34	20	5	7	12
		LRB35	20	7	3	10
		LRB36	20	6	6	12
Montpellier (M)	Inbred lines	MP41	18	6	9	15
		MP42	20	5	2	7
		MP43	6	3	3	6
		MP44	42	4	4	8
		MP45	7	5	2	7
		MP52	45	3	7	10
		MP53	38	5	5	10
		MP54	28	5	7	12
		MP55	61	5	6	11
		MP61	9	3	3	6
		MP62	53	4	5	9
		MP63	5	0	2	2
	Control lines	MPB21	20	3	5	8
		MPB22	20	4	6	10
		MPB23	20	6	4	10
		MPB24	20	3	5	8
		MPB25	20	0	5	5
		MPB26	20	5	5	10
		MPB31	10	4	1	5
		MPB32	10	6	4	10
		MPB33	10	4	1	5
** **	** **	**Total**	** **	**156**	**157**	**313**

### Wing size measurement

Two wings from each mosquito individual were fixed on a glass slide within 50 μl of Eurapal (Carl Roth, Germany). Pictures of the wings were taken under a stereomicroscope x 20 (Leica, Germany) and processed with the Leica LAS software (Leica, Germany). The wings size was estimated for one wing per individual from the intersection of the 2^nd^ and 3^rd^ vein to the intersection of the 7^th^ vein and the apex of the wing (S1 Fig) with the imageJ software (https://imagej.nih.gov/). A total of 43 individuals out of the 313 presented a deterioration of their wings and were referred as missing data points (NA) in the database (S1 Table).

### Mosquito midgut dissection

Individuals were rinsed 3 times with sterile 1X PBS (GIBCO, USA), surface disinfected 5 min in 70% ethanol and rinsed 5 times in 1X PBS. Surface disinfected mosquitoes were dissected under a flow hood with sterile material and appropriate equipment to avoid any potential contamination. The midgut was extracted from the abdomen with forceps under a stereomicroscope. Midgut and carcasses from adult individuals were collected in 100 μl of 1X PBS, within separate tubes.

### Genotyping

Carcasses from individuals were crushed with a sterile pestle in 150 μl of 1X TE solution containing 0.2 mg.ml^-1^ of Proteinase K (Qiagen, Germany). The mixture was incubated 2 h at 57°C, 3 min at 95°C and 2 min at room temperature. After centrifugation 7 min at 16,100 g, 100 μl of supernatant was collected to constitute the DNA sample. The three microsatellite markers Alb-di-6, Alb-tri-3 and Alb-tri-45 were selected for mosquito genotyping based on our previous study showing clear profiles without any stuttering pattern and a low rate of null alleles [24,32]. The PCR primers were respectively Alb-di6F (5' ATTO565-TCT TCA TCT ACG CTG TGC TC 3’), Alb-di6R (5’ GAC GCC AAT CCG ACA AAG TC 3’); Alb-tri3F (5' Yakima Yellow- AGA TGT GTC GCA ATG CTT CC 3’), Alb-tri3R (5’ GAT TCG GTG ATG TTG AGG CC 3’) and Alb-tri45F (5' ATTO565- TTT CAG CTC GGT GTT ATG GC 3’), Alb-tri45R (5’ TGA TGT TGA TGA TGA TGA CTA CGA 3’). PCR mix was performed with Qiagen Type-it Microsatellite PCR Kit following the manufacturer's recommendations and 1 μl of 1/5^th^ diluted DNA of each individual sample. Amplifications were performed as previously described [32]. The PCR products were diluted with a ratio of 1/60 and 1 μl of the dilution was mixed with 13.8 μl of ultrapure Hi-Di-formamide TM and 0.2 μl of size marker (MRL 500). The solution was loaded on an ABI Prism 3730XL Genetic Analyzer automated sequencer (Life Technologies, USA). The microsatellites were manually scored with Genemapper 4.0 (Life Technologies, USA). The genetic diversity was then estimated with FSTAT 2.9.3.2.

### Bacterial Automated Ribosomal Intergenic Spacer Analysis (b-ARISA)

Genomic DNA was extracted from individual midguts following our optimized protocol previously published [24]. To amplify the intergenic region flanking the 16S rDNA and 23S rDNA genes in eubacteria, PCR were conducted with the primers ITSF (5’FAM–GTC GTA ACA AGG TAG CCG TA-3’) and ITSReub (5’-GCC AAG GCA TCC ACC-3’) [33]. The reaction mixture contained 500 nM of each primer, 200 μM of dNTP, 1X of Q5 buffer (New England Biolabs, USA), 1X of High-GC enhancer (New England Biolabs, USA), 0.12 mg.ml^-1^ of Bovine Serum Albumin (New England Biolabs, USA), 0.06 mg.ml^-1^ of T4 gene 32 (New England Biolabs, USA), 0.7 Units of Q5 polymerase (New England Biolabs, USA) and 30 ng of DNA in a final reaction volume of 25 μl. The amplification cycles started with 3 min of denaturation at 94°C followed by 30 cycles with 45 s at 94°C, 1 min at 55°C and 1 min 20 sec at 72°C and an additional amplification step of 1 min 20 sec at 72°C. Each individual sample was amplified in triplicate and pooled. The pooled mixture amplifications were controlled on 1% agarose gel electrophoresis with positive and negative controls. The pooled amplicons were purified using the QIAquick PCR purification kit (Qiagen, Germany), quantified with Nanodrop and diluted at 10 ng.μl^-1^. A total of 8 μl of PCR products was mixed with 6.8 μl of ultrapure Hi-Di-formamide TM and 0.2 μl of size marker (GS-1200 LIZ). The solution was loaded on an ABI Prism 3730XL Genetic Analyzer automated sequencer (Life Technologies, USA) in 96 well plates. A total of 4 96 well plates were used for the experiment. Different plates might provide different intensities and a slight shift in the ARISA profiles. Those are partially controlled by the broad range size marker but should still be considered. As the limited DNA quantity obtained from individual mosquitoes’ midguts did not allow us to replicate the ARISA measurement per individual, the samples were randomly distributed among the plates to partial out this effect from further statistical analysis. The fluorograms were analyzed with Genemapper 4.0 and selected within a range of 100bp–1000bp. The fluorescence picks areas were binned into 5 bp windows with a shift of 1 bp and transformed into Relative Fluorescence Intensity $RFI=\frac{Pick\;area}{\sum_{i=1}^{n}Pick\;area}$ following the previously published method [34].

### Data analysis

Reduction in genetic diversity within the inbred lines was assessed for each cohort based on the expected heterozygosity (*He*). To test the effects of population origin and inbreeding on the *He* levels, we used a beta regression and a likelihood ratio test for nested models comparisons with the R packages *betareg* and *lmtest* [35,36]. The midgut’s bacterial α (Richness and Shannon index) and β (Bray-Curtis dissimilarity) diversity indices were estimated with the *vegan* package in R [37]. The comparative analysis of the microbiota α-diversity was only based on the Shannon index (H’) which reflects the uncertainty to sample similar bacterial Operational Taxonomic Units (OTU) out of a given individual. This value is directly impacted by the number of taxa (richness) and their relative abundance (evenness). H’ is less affected than richness by technical bias (underestimation of the OTU numbers, multiple picks) and comparatives studies showed that high-throughput sequencing and ARISA approaches performed on the same samples presented a strong correlation for this index [38]. To account for fixed (number of individuals, size, sex, line, origin) and random (cohort, plate) variables, the α-diversity variations were analyzed after fitting the values with General Additive Mixed Models (GAMM) and the parameters were tested with an ANOVA. GAMM were preferred to Linear Mixed Models (LMM) as the residuals were non-normally distributed. Therefore, GAMM enabled a smooth pattern in the relation between response and fixed variables, modelling non-linear relationships. The best model was estimated with the Generalized Cross Validation method which associates penalties to the smooth terms [39]. The individual samples dissimilarity was calculated based on the Bray-Curtis index that is ranked from 0 (two individual samples are identical) to 1 (two individual samples are different). For detection of shifts in the β-diversity, a Canonical Analysis of Principal Coordinates (CAP) was conducted. CAP is a multivariate analysis of the dissimilarity which maximizes the separation of individual samples according to continuous or factorial explanatory variables [40]. This method allows the observation of clustering patterns which might be hidden in unconstrained ordinations. Significant explanatory variables were selected by a stepwise process (*ordistep* function). This selection process is based on permutational multivariate analysis of variance (PERMANOVA) successive to the addition or subtraction of the variables. A total of 999 permutations were used and constrains were applied on the permutations to account for technical bias (plate effect) and nested design (cohort). Each variable that significantly influences the β-diversity was kept in the final ordination model. A permutation test was conducted to determine the significance of the fitted ordination according to the recommendations of the *vegan* package [37]. The fixed factors which showed a strong collinearity (R2 > 0.5 or R2 < -0.5) were analyzed separately (S2 Table).

## Results

### Control of genetic diversity reduction in inbred lines

**Fig 2:**
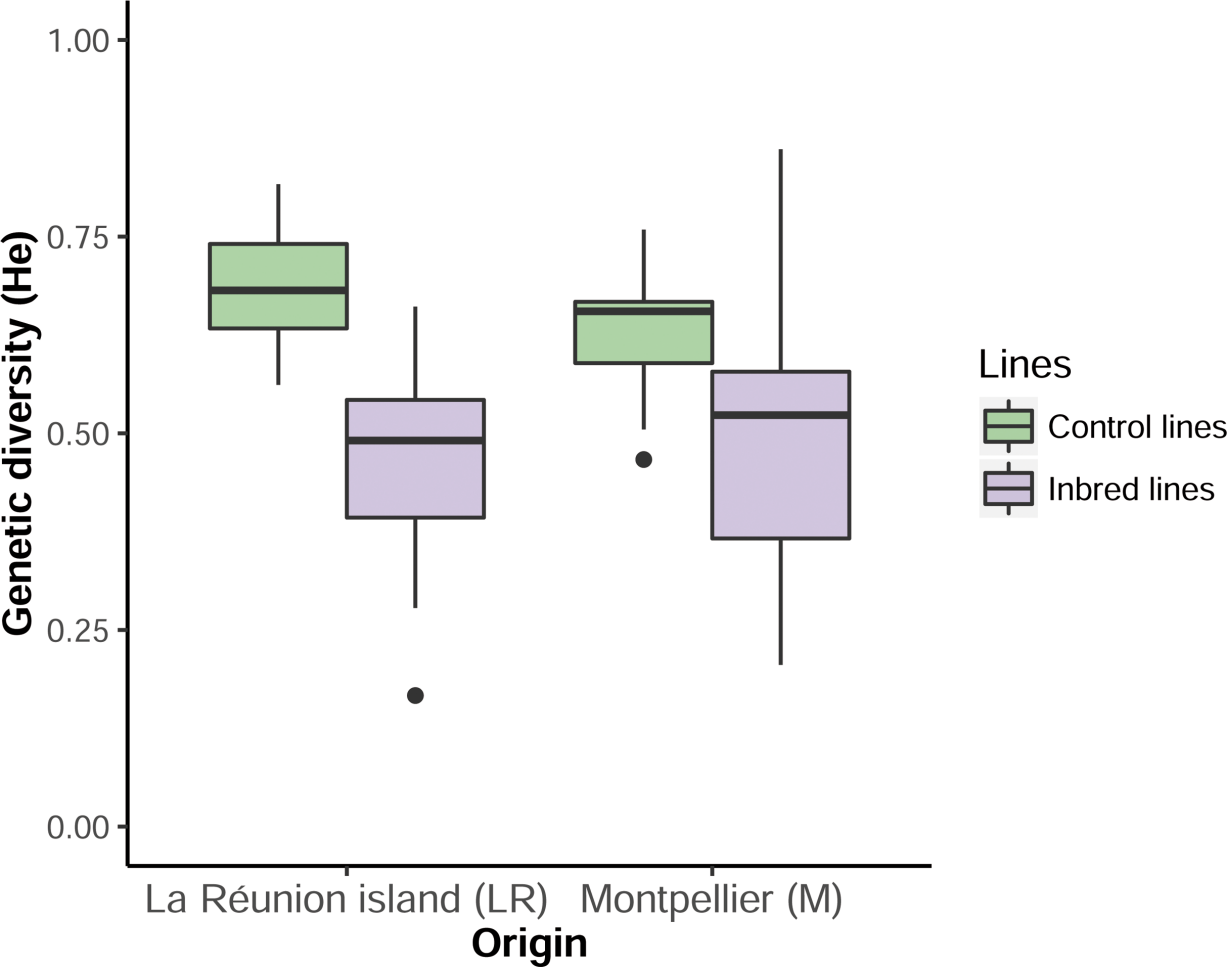
Genetic diversity reduction in inbred lines of mosquitoes. Boxplot of the *He* index after controlled mating of the lines (inbred, control) from the two origins (MP = Montpellier, LR = La Réunion Island).

Female *Ae*. *albopictus* has the ability to store and use sperm from different males [41]. To confirm that such behavior did not affect our aim to reduce the genetic diversity, a control of average *He* index for three microsatellite markers was performed for each cohort. Overall, 18, 5 and 5 alleles were observed for the three markers Alb-di-6, Alb-tri-3 and Alb-tri-45 respectively. *He* index did not differ among individuals from the different origins (*χ*^*2*^ = 1.26; df = 1; p-value = 0.26) (Fig 2). A significant reduction of the genetic diversity (*χ*^*2*^ = 7.80; df = 1; p-value = 0.005) was observed in the inbred lines compared to control ones even though their *He* index was more variable (*χ*^*2*^ = 13.09; df = 1; p-value = 2.97 x 10^−4^) than control lines (Fig 2).

### Impact of origin, genetic diversity and individual factors on the microbiota α-diversity

**Fig 3:**
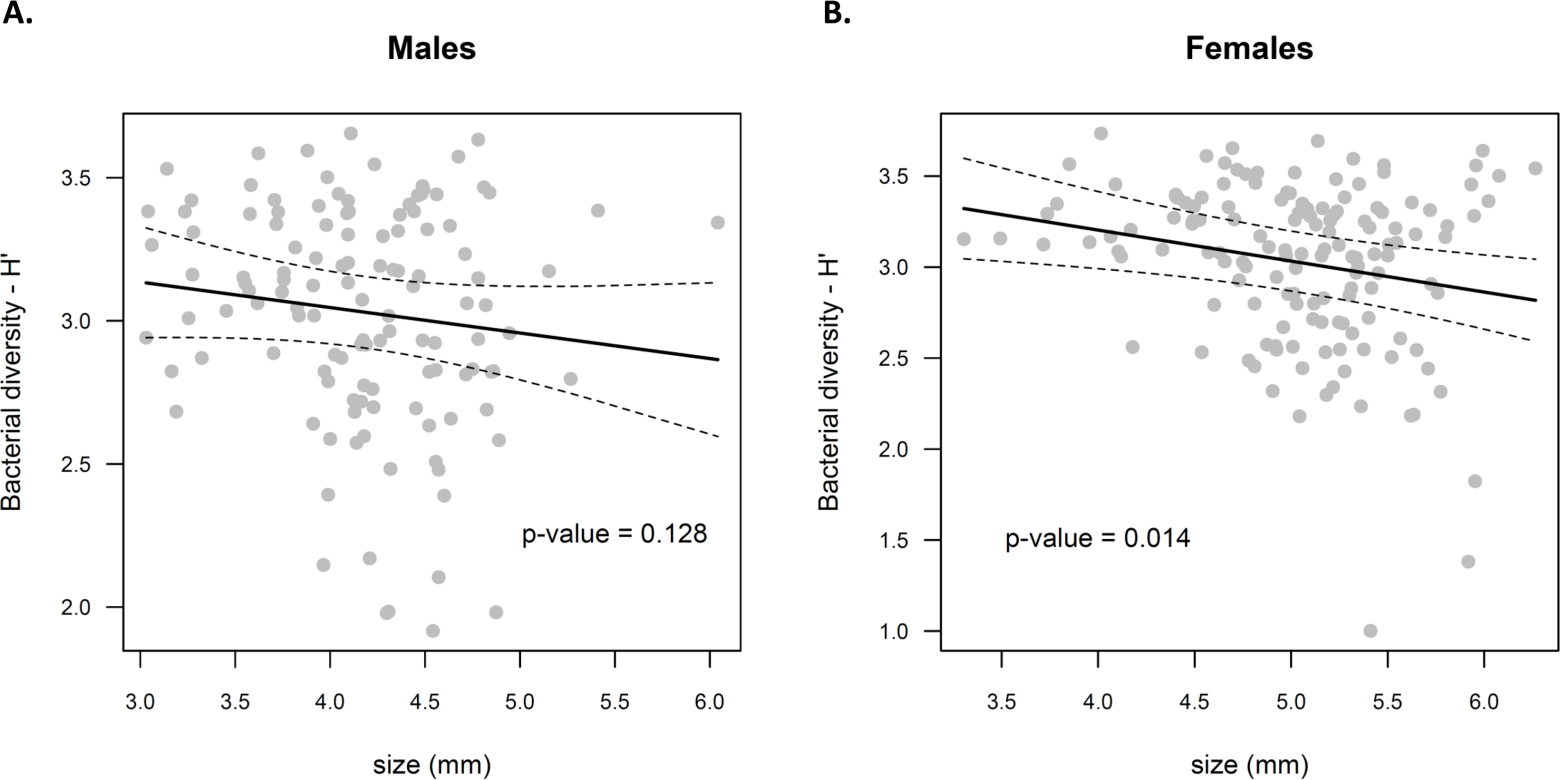
Relationship between mosquito size and the midgut bacterial α-diversity. Fitted GAMM model (solid line) and its standard errors (dashed lines) are represented for (A) Male and (B) female samples.

Operational Taxonomic Units (OTU) represent the ITS variants found within the individual samples. An average of 50.3 ± 12 OTUs was identified within the mosquito midguts. The mosquito lines, population origin, sex and number of individuals per cohort did not influence the *H’* index (Table 2). As sex is collinear with size (R^2^ = -0.62), the size effect might also be linked to the difference in size between sexes. Therefore, the size was only considered while males and females were analyzed separately. It appeared that the size of the individuals has a significant impact on the alpha diversity of females (F = 6.22; df = 1,139; p-value = 0.014) but this effect was not observed on the alpha diversity of males (F = 2.35; df = 1,120; p-value = 0.128) (Table 2, Fig 3). A lower bacterial alpha diversity was observed in larger females.

**Table 2:** Effects influencing the variation of the microbiota α-diversity (shanon index).

Model	Model components	Factors	df / edf*	F	p-value**
**Full model**	**Fixed effects**				
	Parametric terms	Line	1	0.37	0.545
		Population origin	1	0.003	0.959
		sex	1	0.08	0.776
	Smooth terms (approximate significance)	Nb. of individuals per cohort	1	0.67	0.415
**Males only**	**Fixed effects**				
	Parametric terms	Line	1	0.06	0.808
		Population origin	1	0.001	0.970
		Nb. of individuals per cohort	1	0.31	0.580
	Smooth terms (approximate significance)	Size	1	2.35	0.128
**Females only**	**Fixed effects**				
	Parametric terms	Line	1	1.20	0.28
		Population origin	1	0.03	0.872
	Smooth terms (approximate significance)	Nb. of individuals per cohort	1	0.09	0.761
		Size	1	6.22	**0.014**

* degree of freedom (Parametric terms) or estimated degree of freedom (Smooth terms)

** The plate and cohort were used as a random effect

### Impact of origin, genetic diversity, and individual factors on the microbiota β-diversity

**Fig 4:**
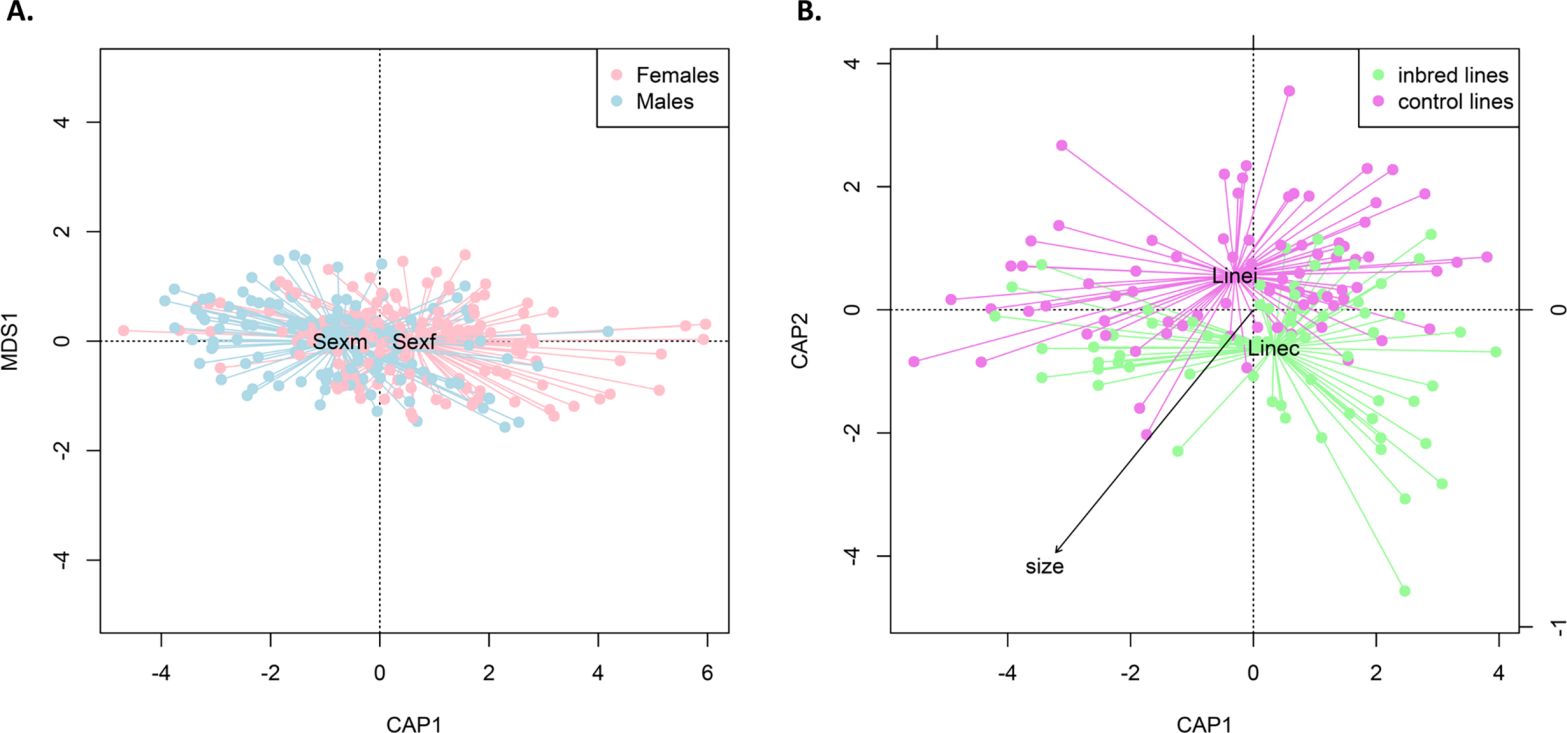
Canonical Analysis of Principal Coordinates (CAP) of the midgut bacterial β-diversity among mosquito populations. (A) The full dataset has been used and the CAP represents the impact of the mosquito sexes (Sexf and pink colors = Females, Sexm and blue color = Male) on the Bray-Curtis dissimilarities values among individual midguts with non-random structures (F_PERMANOVA_ = 1.89; df = 1,265; p-value = 0.027). (B) Only the females’ dataset has been used and the CAP represents the impact of the mosquito lines (linei and purple = inbred, linec and green = control) on the Bray-Curtis dissimilarities values among individual midguts with non-random structures (F_PERMANOVA_ = 1.46; df = 2,139; p-value = 0.038).

On average, the Bray-Curtis dissimilarity was 0.64 ± 0.15. Once constrained for the technical biases (plate), the cohort effect explained 20% of the similarity (F_PERMANOVA_ = 0.22; df = 34,160; p-value = 0.001). Such result suggests a strong impact of identical cohort rearing. CAP analysis was performed with a correction for cohort and plate effects. Stepwise selection resulted in the conservation of sex (F_PERMANOVA_ = 1.89; df = 1,265; p-value = 0.027). This final model included one term and significantly represents non-random variations in *Ae*. *albopictus* midgut microbiota (Fig 4A). Even if the sex of the mosquitoes significantly shaped the bacterial communities structure of *Ae*. *albopictus*, it only explained 0.71% of the dissimilarity variation. Furthermore, due to the detected collinearity between size and sex (R^2^ = -0.62), the analysis was performed separately for the males and the females. In females, the stepwise permutational analysis enabled the selection of two factors namely size (F_PERMANOVA_ = 1.62; df = 1,139; p-value = 0.04) and line (F_PERMANOVA_ = 1.51; df = 1, 139; p-value = 0.02) that correlated with a shift in the microbiota community structure (Fig 4B). However, none of the factors impacted the β-diversity of males’ microbiota. Indeed, the best model for the males only included the size effect which was not significant (F_PERMANOVA_ = 1.60; df = 1, 120; p-value = 0.06).

## Discussion

Insects’ gut is a very selective habitat for microorganisms due to lytic enzymatic activities and immune response, as well as extreme pH conditions (alkaline in the case of mosquitoes) and molting [42]. Intraspecific comparisons of insect gut microbiota composition showed a consistent pattern of divergence according to host habitat, food and phylogeny [43]. At the intraspecific level, insect gut microbiota can also be driven by vertical and horizontal microorganism transmissions which will tend to homogenize the microbial communities within hosts’ populations [42]. Several studies reported divergences in the bacterial communities structure associated with local populations of mosquitoes (local group of individuals from the same species) [12]. Environmental factors or genetic background of the populations could partly explain such divergences. The latter would particularly be true in the case of vertically transmitted symbionts which often coevolve with their host and are also more likely to spread locally if they reach a certain prevalence threshold [44]. Our recent study, focusing on invasive and native populations of the Asian tiger mosquito *Ae*. *albopictus*, lacked to highlight any correlation between host genetic diversity and bacterial microbiota structures but demonstrated a consistent correlation between host genetic diversity and bacterial diversities [24]. To assess a possible impact of the host genetic diversity on mosquito associated bacterial microbiota diversity, we conducted an experimental study with laboratory mosquito populations by removing environmental factors susceptible to co-variate with the mosquito genetic structure and diversity. Mosquito populations were collected in La Réunion (an old tropical population isolated on an island in the Indian Ocean) and Montpellier (a recent temperate invasive population from the Mediterranean coast) [29].

When reared in laboratory conditions, no differences in the microbiota diversity were observed between those populations. An artificial reduction of the host’s genetic diversity in both populations did not highlight any variation in the α diversity of *Ae*. *albopictus* midgut bacterial communities. These observations reject the hypothesis that within line diversity in microbiota composition is driven by host genetic variation. Our previous investigation of *Ae*. *albopictus* midgut microbiota field populations from France and Vietnam showed a relative similarity among those origins due to the maintenance of the dominant symbiont *Dysgonomonas* sp. within all the populations [24]. However, in that case, marginal variations were observed among the origins and those correlated with their genetic diversity and were confounded with environmental factors. In our study, the genetic diversity *per se* seems to be correlated with a shift in the microbiota of females but not in males. This result would suggest that the increasing of genetic similarity between individuals reared in a similar container induce a shift in their associated microbial community. Such a shift might be driven in favor to vertically transmitted symbionts which are often transmitted by females [44]. Despite this apparent effect, we cannot reject a potential genotype by environmental interaction. Indeed, the laboratory-reared mosquitoes might have lost a part of their natural microbiota community. Such effects were notably demonstrated by a field controlled study based on roots and shoots microbiota of different wild mustard genotypes [45]. In addition to genotype by environment interactions, specific genotype by genotype interactions may also occur between hosts and specific microbes. Those interactions are unlikely to be revealed by our protocol that focuses on global community associations. Such genetic associations between host and microbes have been described in several models. Genome-wide associations (GWA) and targeted experiments conducted with mutant lines of *Drosophila melanogaster* have shown a significant association between hosts genes and *Acetobacter tropicalis* [46]. On the symbiont side, GWA studies revealed the importance of type IV pili, amino acid synthesis and iron intake genes in the bee hindgut colonization ability of the symbiotic bacterium *Snodgrassella alvi* [47]. Similarly, GWA conducted on mammals, revealed discrete host candidate genes [48].

Our study demonstrated a correlation between the forewing length (size) and midgut bacterial diversity which was consistent for females. Indeed, an increase in size was associated with a decrease in the midgut bacterial diversity and a shift in the microbiota composition of females. Previous studies focusing on captive mosquitoes demonstrated that the wing size was strongly and positively correlated with the adults body weight [49–51]. Mosquitoes sizes have also been shown to be driven by immature developmental conditions [52–55]. Therefore, we could assume that individual variations in developmental conditions among female mosquitoes might have led to different community assembly. Such hypothesis would also be consistent with the high contribution of the cohort factor to the bacterial community dissimilarity. Indeed, several studies reported the colonization of the gut of mosquitoes by habitat-related symbionts (e.g. *Cyanobacteria* in larvae) [56,57].

The wing size–bacterial community correlation could also be the indirect developmental response of the mosquito to specific bacterial colonization. Indeed, axenic *Ae*. *atropalpus* larvae re-infected with different native and non-native bacterial symbionts reach smaller or equal sizes to the control group (undisturbed microbiota) [58]. Similarly, *Ae*. *aegypti* larvae challenged with the pathogenic bacterium *Bacillus thuringiensis* var. israelensis develop into smaller adults [59]. It remains difficult to emphasize whether such interaction would occur in *Ae*. *albopictus*. Indeed, challenges of *Ae*. *aegypti* or *Culex pipiens* with several bacterial symbionts did not impact the individuals’ development [58,60]. If the symbionts have an impact on the mosquitoes’ size, such interactions would impact populations dynamics since the size of *Ae*. *albopictus* individuals is related to their fecundity and survival [61,62].

Due to their importance in human and animal health, the global mosquito literature remained strongly biased over female studies. In this study both sexes were compared and our data support a small but significant structuration of the gut bacterial microbiota among sexes. In the current studies, none of the adults have been fed before collection and no dispersion occurred. Therefore, none of the divergences between the sexes could be related to food or individual’s dispersal. Male and female associated microbial communities of mature or field collected mosquitoes are often confounded with the nutritional and behavioral habits of both sexes. Males only feed on plant material (nectar, fruit) and are poorly dispersing, whereas females also feed on vertebrate blood and disperse around the breeding sites. Reproductive organs of mosquitoes (testes and ovaries) are specifically colonized by different bacterial communities [63]. This is also the case for *Ae*. *albopictus*, as its dominant symbionts *Wolbachia w*AlbA and *w*AlbB mainly colonize females ovaries [26]. Due to their vertical transmission through females, those bacteria reached higher densities in females than in males [64–66]. However, the effects that have been observed here are unlikely to reflect variations in *Wolbachia* densities within the midgut due to their poor ability to colonize digestive tissues [24,26] and the low *Wolbachia* densities in early adults which did not show any sex related differences at this stage [64,67]. It is important to specify that our study was conducted on unfed freshly emerged adults. Given that the microbiota is drastically reduced right after the pupal stage [12], it is possible that larvae or mature adults harbor a different community and respond differently to the factors considered here.

## Conclusion

In this study, we showed an absence of relationship between population genetic bottleneck, origin and midgut microbiota associated with *Ae*. *albopictus*. Consequently, we suggest that the bacterial communities are poorly structured by the genetic background or diversities of the host populations. However, both sexes harbored a different bacterial microbiota. In addition, those bacterial communities co-varied with female sizes. They should be investigated with more details to decipher the underlying mechanism of such symbiotic interactions. Since the similarity within cohort was high, we also suggest that individual rearing conditions could be of main importance to shape adult microbiota. Future investigations including environmental covariates on1 top of the rearing conditions should be performed to catch the effects of such larval habitat quality on the sustainable colonization of microbes within larvae.

## Supporting information

S1 Fig Wings length measurements. The length was measured between two landmarks which correspond to (l1) the intersection of the 2nd and the 3rd vein as well as (l2) the intersection of the 7th vein and the wing border.
(PDF)

S1 Table Dataframe. (TXT)

S2 Table Pearson correlation among the fixed factors. (XLSX)

S3 Table Alpha diversity and size summary. (XLSX)
