# Supplementary material for: Shared larval rearing environment, sex, female size and genetic diversity shape Ae. albopictus bacterial microbiota
Source: PLoS One. 2018 Apr 11;13(4):e0194521. doi: 10.1371/journal.pone.0194521 (PMC5894977; doi:10.1371/journal.pone.0194521)
Supplement: S1 Fig — The length was measured between two landmarks which correspond to (l1) the intersection of the 2nd and the 3rd vein as well as (l2) the intersection of the 7th vein and the wing border. (PDF) [file pone.0194521.s001.pdf]

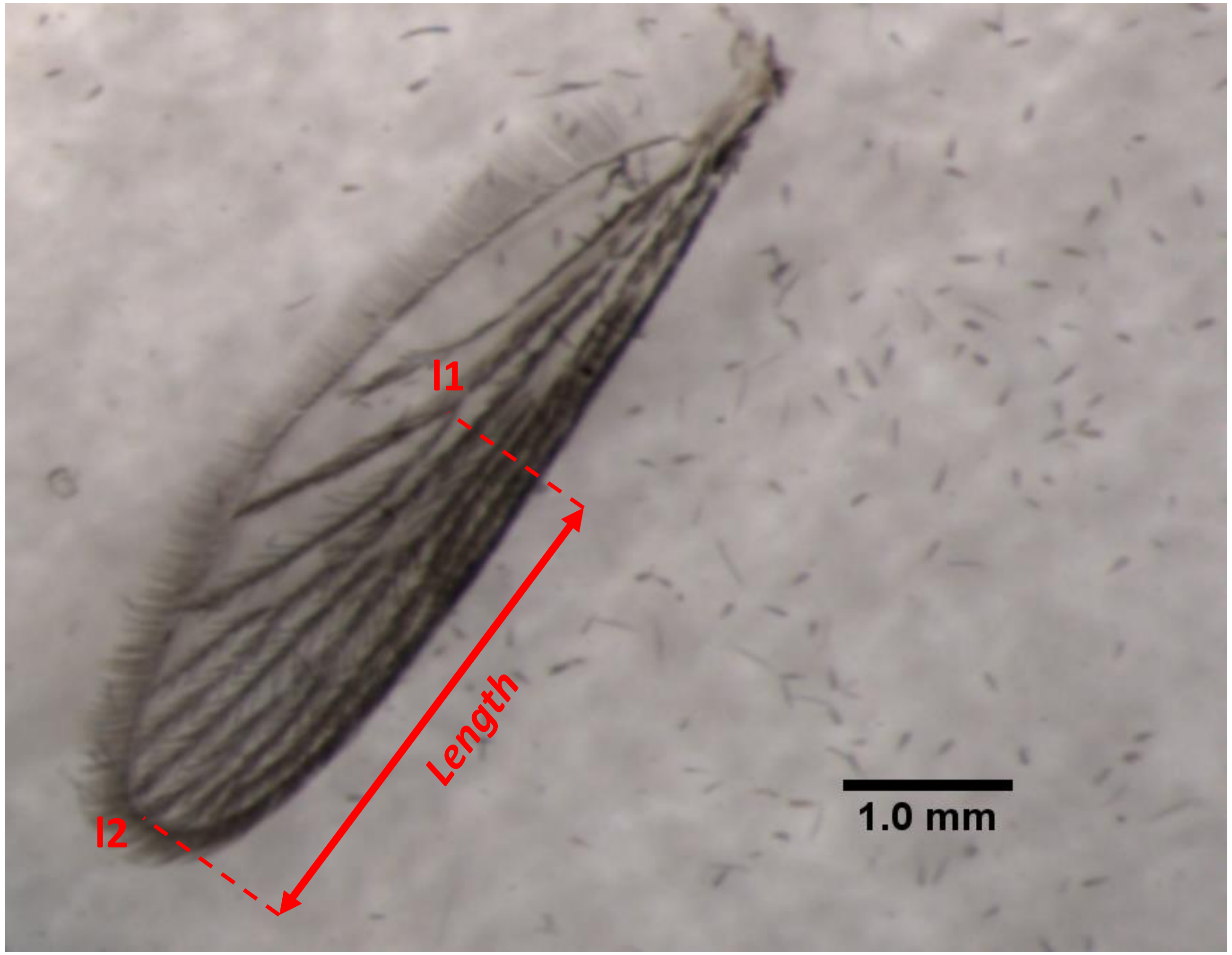

Figure S1. Wings length measurements. The length was measured between two landmarks which correspond to (l1) the intersection of the 2nd and the 3rd vein as well as (l2) the intersection of the 7th vein and the wing border.
